# Efficacy of a novel double-controlled oncolytic adenovirus driven by the Ki67 core promoter and armed with IL-15 against glioblastoma cells

**DOI:** 10.1186/s13578-020-00485-1

**Published:** 2020-10-27

**Authors:** Qing Zhang, Junwen Zhang, Yifu Tian, Guidong Zhu, Sisi Liu, Fusheng Liu

**Affiliations:** 1 Brain Tumor Research Center, Beijing Neurosurgical Institute, Capital Medical University, Beijing, 100070 P.R. China; 2 Department of Neurosurgery, Beijing Tiantan Hospital Affiliated to Capital Medical University, Beijing, 100070 P.R. China; 3 Beijing Laboratory of Biomedical Materials, Beijing, 100070 P.R. China; 4 Department of Ophthalmology, Peking University People’s Hospital, Eye Diseases and Optometry Institute, Beijing Key Laboratory of Diagnosis and Therapy of Retinal and Choroid Diseases, College of Optometry, Peking University Health Science Center, Beijing, P.R. China

**Keywords:** Glioblastoma, Oncolytic adenovirus, Ki-67 promoter, IL-15, Immunotherapy, Angiogenesis

## Abstract

**Background:**

Glioblastoma (GBM) is an immunosuppressive, highly vascular and devastating malignant brain tumor. Even with progressive combination treatment that includes surgery, radiotherapy, and chemotherapy, the prognosis for GBM patients is still extremely poor. Oncolytic adenovirus (OAd) can specifically replicate in GBM cells, permitting the rapid copy of the therapeutic genes it carries. Moreover, E1A is an essential gene in adenoviral replication and is the first gene expressed upon viral infection. E1A expression can be regulated by the Ki67 promoter, while the CMV promoter drives therapeutic gene expression. However, the efficacy of a double-controlled OAd driven by the Ki67 core promoter and armed with IL-15 against GBM cells has not been investigated.

**Methods:**

Fluorescence microscopy was performed to evaluate infection ability in the viruses. Cell viability was detected by CCK-8 assay. Levels of cytokines in different supernatants were determined by ELISA, and IL-15 gene expression was measured by RT-PCR. Angiogenic capacity was analyzed by tube formation assay.

**Results:**

We successfully constructed a double-controlled oncolytic adenovirus driven by the Ki67 core promoter and armed with IL-15 that selectively infected and killed GBM cells while sparing normal cells. The adenoviruses prime IL-15 gene expression to significantly enhance anti-GBM efficacy through effective activation of microglial cells. Moreover, OAd not only directly inhibits angiogenesis but exhibits potent antiangiogenic capacity mediated by the reduction of VEGF secretion.

**Conclusions:**

These results provide new insight into the effects of a novel double-controlled OAd driven by the Ki67 core promoter and armed with IL-15 in glioblastoma treatment, which may help in the development of novel therapies in solid tumors.

## Introduction

Glioblastoma (GBM) remains a refractory and lethal disease despite decades of comprehensive research. GBM expresses a variety of proteins that bind to T cell surface receptors, leading to T cell dysfunction and apoptosis [1, 2], and GBM microenvironment signals, such as TGF-β and IL-10, induce local and systemic immunosuppression [3]. Despite the introduction of concomitant and adjuvant radiotherapy and chemotherapy, patient prognosis remains unsatisfactory, with an almost 15 months median survival [4–6]. These poor outcomes are partially linked to extreme degrees of genetic and phenotypic variation, as well as therapeutic resistance [7]. Therefore, novel approaches are urgently needed to improve prognosis in glioma patients.

Oncolytic adenovirus (OAd) is one of the newly developed methods for the treatment of glioma that can selectively infect and promote lysis of glioma cells while sparing normal tissues [8]. OAd is currently one of the most widely used carriers that offers several advantages over other therapies, including an intrinsic ability to kill infected cells at the completion of the viral replication cycle and the capacity to deliver therapeutic transgenes [9]. Despite oncolytic viruses having some potential pitfalls for glioma treatment [10], an increasing number of studies on OAd that express immunomodulatory transgenes in glioma have yielded beneficial outcomes. OAds that expressed the immune costimulator OX40L exhibited inhibition of gliomas and significantly increased survival through tumor-specific activation of lymphocytes and proliferation of CD8^**+**^ T cells [11]. OAd armed with IL-4 also showed potent anti-glioma immune activity in several glioma models [9]. Adenoviral E1A, the first gene expressed upon oncolytic adenoviral infection, plays a crucial role in viral replication [12]. To improve specific anti-tumor activity of OAd, many researchers have used tumor-specific promoters to regulate the adenoviral E1A gene and design novel OAds, which can be controlled to proliferate in tumor cells and have high safety, utilizing tumor-specific promoters to drive E1A expression [12–14].

Ki-67 is a nuclear protein that is closely associated with cellular proliferation and the cell cycle in tumors [15]. Ki-67 is also a classic proliferation markers in human glioma. Researchers have found that the Ki-67 expression phenotype is associated with distinct changes in gene expression associated with the regulation of cell growth and proliferation [16, 17]. Background levels of Ki-67 expression in the normal brain are very low, and Ki-67 levels are correlated with higher glioma grade and poor prognosis. The dismal prognosis of GBM patients is correlated with an increased Ki67 proliferation index [18, 19]. Therefore, differential Ki67 gene expression in glioma tissue compared with normal tissue provides an opportunity for the design of a Ki67 promoter-controlled OAd. The Ki-67 gene promoter is an excellent tumor-selective promoter with the desirable specificity and efficiency to further control transgene expression within tumor cells and to improve targeting in gene therapy.

IL-15 is a micromolecular protein and member of the 4-alpha-helix bundle family of cytokines that is an immune-regulatory factor with very similar functions to IL-2 [20]. Despite the fact that it is related to IL-2, IL-15 possesses contrasting roles in adaptive immune responses. It has been well documented that IL-15 plays a crucial role in immune cell function [21, 22]. Furthermore, immune cells activated by IL-15 have been shown to have a high capacity for cytokine secretion, including IFN-γ, TNF-α, TNF-β, GM-CSF, and IL-10, resulting in apoptosis of tumor cells [23, 24]. Previous studies demonstrated that IL-15 enhances the cytolytic activity of natural killer cells, CD8^+^ T cells, and natural killer T cells, as well as the development of macrophage and neutrophil immune response [25, 26]. For the sake of utilizing the enhanced anti-tumor capacity of IL-15, we inserted the IL-15 gene into the CMV promoter in the E3 region to control its expression.

In this study, we developed a replication-competent OAd regulated by the Ki67 core promoter and armed it with IL-15. We demonstrated that the novel double-regulated OAd expressing IL-15 produced an enhanced anti-tumor effect against GBM cells. Moreover, we demonstrated that OAd showed strong anti-angiogenic capacity by decreasing VEGF secretion from glioma cells. This research is innovative because the therapeutic efficacy of a dual-regulated OAd armed with IL-15 for glioma has not been designed or tested to date.

## Materials and methods

### Isolation and culture of human glioblastoma cells

Human brain tumor samples were obtained from the Neurosurgery Department at Tian Tan Hospital in Beijing, China, after patients with glioma provided informed consent. Specimens were reviewed by a neuropathologist to assess the grade and tumor type before assays were performed. Human glioma samples were transferred to a Petri dish, washed three times in phosphate-buffered saline (PBS, Gibco, USA), and cut into 1-mm^3^ pieces. Next, 0.05% trypsin (Gibco, USA) was added to the tumor specimens. Then, the single pieces were digested for 10 min, filtered with a 70-µm nylon mesh (Corning, USA) and centrifuged at 1000 rpm for 5 min. Single cells were resuspended in Dulbecco’s Modified Eagle’s Medium (DMEM) (Gibco, USA) containing 10% fetal bovine serum (FBS; Invitrogen, China) and 100 U/ml penicillin/streptomycin (Gibco, USA), seeded into 25-cm^2^ culture flasks (Corning, USA) and incubated at 37 °C and 5% CO_2_ in a humidified chamber. The media was changed 2–3 times a week. Primary cells at 70–80% confluence were passaged using 0.05% trypsin (Gibco, USA) and were used for different experiments after purification. This line of primary GBM cells was named BT-01 and identified by short tandem repeat (STR), moreover, the biological characteristics of the primary GBM cells can be found in our previous study [27].

### Cell lines

GL261, U251, U87, BV2 and HMC3 cells were purchased from the American Type Culture Collection (ATCC, Gaithersburg, MD, USA), and human umbilical vein endothelial cells (HUVECs) were purchased from Lonza (MD, USA). Cell lines GL261, U251, U87, BV2 and HMC3 were cultured in DMEM (Gibco, USA) containing 10% fetal bovine serum (FBS; Invitrogen, China) and 100 U/ml penicillin/streptomycin (Gibco, USA). HUVECs were cultured in RPMI 1640 medium (Gibco, USA) supplemented with 10% fetal bovine serum (FBS; Invitrogen, China) and 1% penicillin/streptomycin (Gibco, USA). All cells were cultured in a 37 °C humidified incubator with 5% CO_2_.

### Recombinant oncolytic adenovirus

The Ki67 promoter gene was amplified and obtained. The virus skeleton plasmids BamHI/XhoI and pTE-Mel containing the GFP gene were successfully constructed by the laboratory at Chinese Academy of Medical Sciences and Peking Union Medical College (Beijing, China). The plasmids were linked with Ki67 promoter to form pTE-Ki67MEl/pSh5-GFP and were treated with incision enzyme Mfe I. pSh5-GFP was linked to pTE-MEl and pTE-Ki67MEl to construct pSh5-MEl-GFP and pSh5-Ki67-GFP, followed by treatment with the restriction enzyme PME I. Then, plasmids were extracted, and pAd5-GFP and pAd5-Ki67/GFP were obtained. Similarly, in order to construct pAd5-Ki67/IL-15, GFP was replaced with the IL-15 gene. The above constructed plasmids were cotransfected with pAd5-GFP and pAd5-Ki67/GFP into 293T cells using Top10. After viral plaque purification, the identified adenoviruses were termed Ad5-GFP, Ad5-Ki67/GFP and Ad5-Ki67/IL15.

### Collection of conditioned media

For all experiments, glioma cells, GL261, U251, and U87, primary cells BT-01, and microglial cells, BV2 and HMC3, were seeded into T25 tissue culture flasks in DMEM with 10% FBS containing penicillin (100 U/ml)/streptomycin (100 mg/ml) (Gibco, Grand Island, USA). After culturing for 24 h, each oncolytic adenovirus (Ad5-GFP, Ad5-Ki67/GFP, Ad5-Ki67/IL-15) at a multiplicity of infection (MOI) of 40 was added separately to the T25 tissue culture flasks, and glioma cells were treated with the viruses for another 24, 48, 72 or 96 h. At various time points, different glioma conditioned media (CM, Ad5-GFP-CM, Ad5-Ki67/GFP-CM and Ad5-Ki67/IL-15-CM) were collected from the flasks and centrifuged at 2000 rpm for 10 min to remove cells and cellular debris. Microglial cells, BV2 and HMC3, were cultured in standard medium, and respectively treated with different viruses (Ad5-GFP, Ad5-Ki67/GFP and Ad5-Ki67/IL-15, MOI = 40) for 12 h, then the BV2 and HMC3 cells were washed three times with PBS to remove the viruses, and continued to be cultured in standard medium for another 72 h, at which time different microglial cells conditioned media (HMC3/BV2-Ad5-GFP-CM, HMC3/BV2-Ad5-Ki67/GFP-CM, and HMC3/BV2-Ad5-Ki67/IL15-CM) were obtained and centrifuged at 2000 rpm for 10 min to remove cells and cellular debris. Afterward, all collected conditioned media were stored at − 20 °C prior to use.

### Fluorescence microscopy

Glioma cells, GL261, U251, and U87, primary cells BT-01, and microglial cells, HMC3 and BV2, were treated with Ad5-GFP and Ad5-Ki67/GFP at a multiplicity of infection (MOI) of 40 and were observed under an Olympus microscope. Images were taken 72 h after infection. GFP expression levels were analyzed in three random fields of view per well using ImageJ software (NIH, USA).

### Cell proliferation assay

Cell viability was analyzed using the Cell Counting Kit-8 (CCK-8 Kit, Dojindo Laboratories, Japan). Glioma cells, GL261, U251, and U87, primary cells BT-01 and BV2, and HMC3 cells were seeded into 96-well plates (3000 cells/well) and cultured overnight. Then, the oncolytic adenoviruses (Ad5-GFP, Ad5-Ki67/GFP, and Ad5-Ki67/IL-15) were added to the 96-well plates at different MOI values and cultured for 72 h. In addition, Ad5-GFP, Ad5-Ki67/GFP and Ad5-Ki67/IL-15 were added to 96-well plates containing glioma cells at an MOI of 40 and cultured for 1, 2, 3, and 4 days, with standard media as a control. Moreover, the conditioned media of virus-treated microglial cells were added to 96-well plates containing glioma cells and cultured for 1, 2, and 3 days, and untreated conditioned media were used for controls. At various time points, 10 µl of CCK-8 was added to each well and incubated for 2 h at 37 °C and 5% CO_2_. Finally, the absorbance of each well was measured at 450 nm using a microplate reader (PerkinElmer, USA). At least three wells were used for each sample in different condition. Assays were repeated at least three times.

### Detection of IL-15 and VEGF by ELISA assay

IL-15 levels in conditioned media collected from Ad5-Ki67/IL-15-treated glioma cells for 24, 48 h, 72, and 96 h were measured using their respective human ELISA kits (Neobioscience, China). VEGF levels in conditioned media collected from virus-treated glioma cells for 72 h were measured using their respective human ELISA kits (Neobioscience, China). All procedures were performed as described in the manufacturer’s instructions, and absorbance was measured at 450 nm. Repeated wells were used for each media sample.

### RNA extraction and RT-PCR

GL261, U251, and U87 glioma cells and primary cells BT-01 were treated with Ad5-Ki67/IL-15 at an MOI of 40. Total RNA was isolated 48 h postinfection from all glioma cells using TRIzol reagent (Invitrogen, USA). Then, qPCR assays were performed using SYBR-Green PCR Master Mix (Applied Biosystems) on a QuantStudio 6 Flex system (Applied Biosystems). cDNA synthesis was performed using the Reverse Transcription System Kit (Promega A3500) according to the manufacturer’s instruction. The codon-optimized IL-15 with the following primers: forward primer, 5′-CATGTACGTTGCTATCCAGGC-3′; reverse primer, 5′-GGTCTTCTCCTCC AGCTCCT-3′. Each target was run in triplicate, and GAPDH was used as an internal standard. Relative gene expression was compared to a housekeeping gene. The results were calculated using the 2^−ΔΔ*C*t^ method.

### Tube formation assay

Growth factor-reduced Matrigel (BD, USA) was added to flat-bottomed, precooled, 96-well plates. After incubation at 37 °C and 5% CO_2_ for 40 min, HUVECs pretreated with different viruses (Ad5-GFP, Ad5-Ki67/GFP and Ad5-Ki67/IL-15, MOI = 40) for 72 h and untreated HUVECs were labeled using Calcein AM (Tocris, USA). Treated HUVECs (2 × 10^4^/well) were seeded into wells containing standard medium. The untreated HUVECs were seeded into wells containing different U251 conditioned media (CM, Ad5-CM, Ad5-Ki67-CM and Ad5-Ki67/IL-15-CM). In addition, human recombinant vascular endothelial growth factor (VEGF, 6 ng/ml, Peprotech, USA) was added to the extra wells containing CM, Ad5-CM, Ad5-Ki67-CM and Ad5-Ki67/IL-15-CM. Then, 96-well plates were incubated at 37 °C and 5% CO_2_. Three wells were used for each media sample. After 6 h, tube formation was imaged with an Olympus microscope. Capillary-like tube formation of HUVECs was analyzed in three random fields of view per well using ImageJ software (NIH, USA).

### Statistical analysis

Statistical analyses were performed using GraphPad Prism. Unless specifically noted, all data are representative of at least three separate experiments. Error bars represent the standard deviations (SDs) and were calculated using Prism. The specific statistical tests used were t-test for single comparisons or ANOVA followed by Tukey’s test for multiple comparisons, and all P-values < 0.05 were considered statistically significant.

## Results

### Construction of novel oncolytic adenovirus

To explore Ad5-Ki67/IL-15’s contribution to augmenting anti-glioma efficacy, we constructed the Ad5-Ki67 using the Ki67 promoter gene instead of the type 5 adenovirus endogenous promoter of the E1A gene, as well as CMV promoter-regulated replication defects adenovirus Ad5-GFP (1 × 10^12^ vp/ml). Based on Ad5-Ki67, we inserted a CMV-regulated GFP gene into the E3 gene region, and the virus was named Ad5-Ki67/GFP (1.3 × 10^12^ vp/ml). Further, the GFP gene region was replaced with the human IL-15 gene, and the virus was able to release IL-15, which acted synergistically to enhance anti-tumor effects through increased activation of immune cells. This new virus was named Ad5-Ki67/IL-15 (1 × 10^12^ vp/ml). A diagram of the recombinant oncolytic adenovirus expressing IL-15 DNA structure is shown in the supplemental material (Additional file 1: Fig. S1).

### Ad5-Ki67/IL-15 induces efficient delivery of IL-15 in GBM cells

**Fig. 1:**
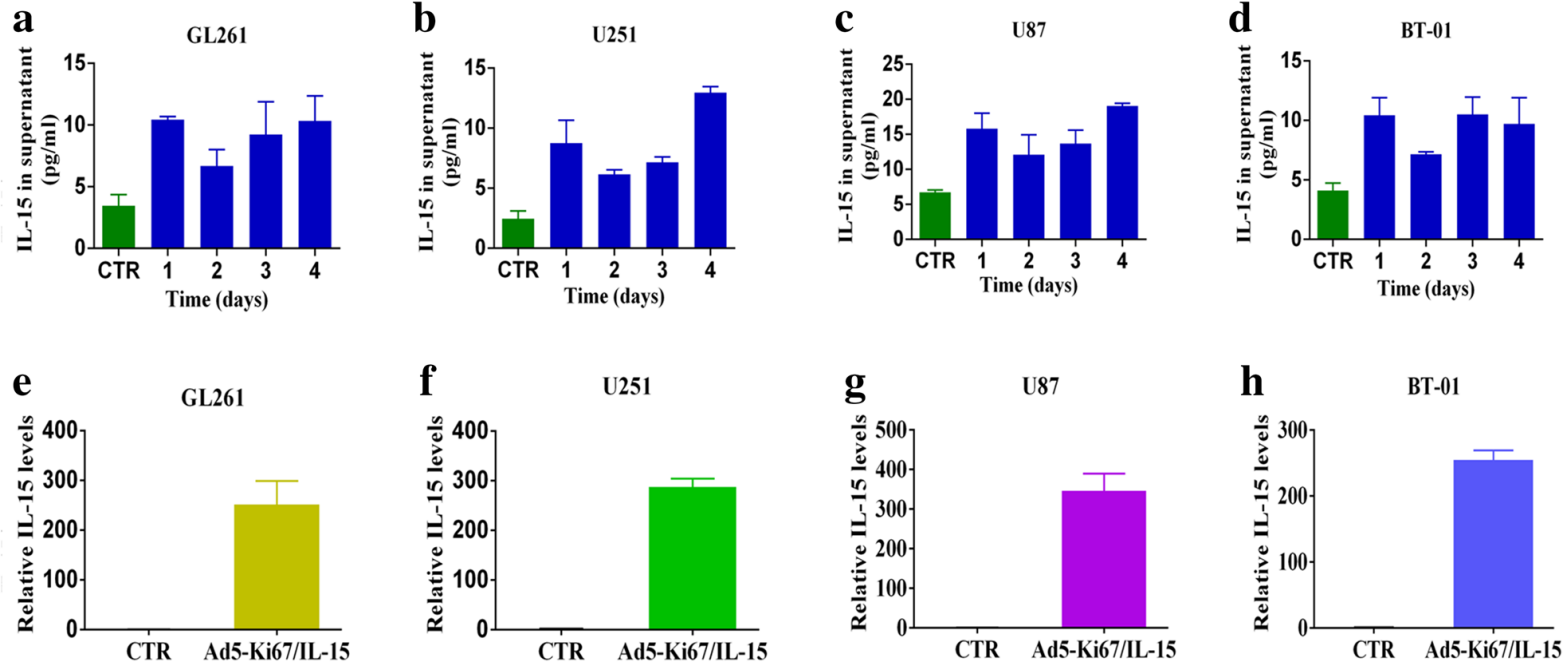
Expression of IL-15 in GBM cells treated with Ad5-Ki67/IL-15. **a**–**d** ELISA was used to measure IL-15 levels in conditioned media that different glioma cells were treated with Ad5-Ki67/IL-15 (MOI = 40) for 24, 48, 72, and 96 h. Untreated conditioned medium was used as a control. **e**–**h** IL-15 gene expression in GL261, U251, U87 and primary cells BT-01 treated with Ad5-Ki67/IL-15 for 72 h by qPCR. Untreated GBM cells were used as a control

To determine whether the recombinant viruses expressed physiologically relevant levels of IL-15, we quantified culture supernatants from Ad5-Ki67/IL-15-treated GL261, U251, and U87 glioma cells and primary cells BT-01 cells using a commercially available ELISA kit specific for IL-15 heterodimers. We found that IL-15 levels were higher in the GL261, U251 and U87 glioma cells and primary cells BT-01 treated with Ad5-Ki67/IL-15 than in the control group after 24, 48, 72, and 96 h. The concentration of IL-15 reached higher values at 96 h after infection of GBM cells, 10.325, 12.97 and 19.32 pg/ml in GL261, U251 and U87 cells, respectively, while primary cells BT-01 appeared to reach a higher value of 9.71 pg/ml 72 h post transfection (Fig. 1a–d), demonstrating that Ad5-Ki67/IL-15 mediates efficient and stable expression of IL-15. Similarly, we also analyzed and found the gene expression of IL-15 was markedly increased in GBM cells after infection with Ad5-Ki67/IL-15 using RT-PCR (more than 200-fold compared to the control group), while lower IL-15 levels were maintained in untreated GBM cells (Fig. 1e–h). Therefore, Ad5-Ki67/IL-15 can regulate IL-15 expression in GBM cells.

### The dual-regulated oncolytic adenovirus selectively infects and kills GBM cells

**Fig. 2:**
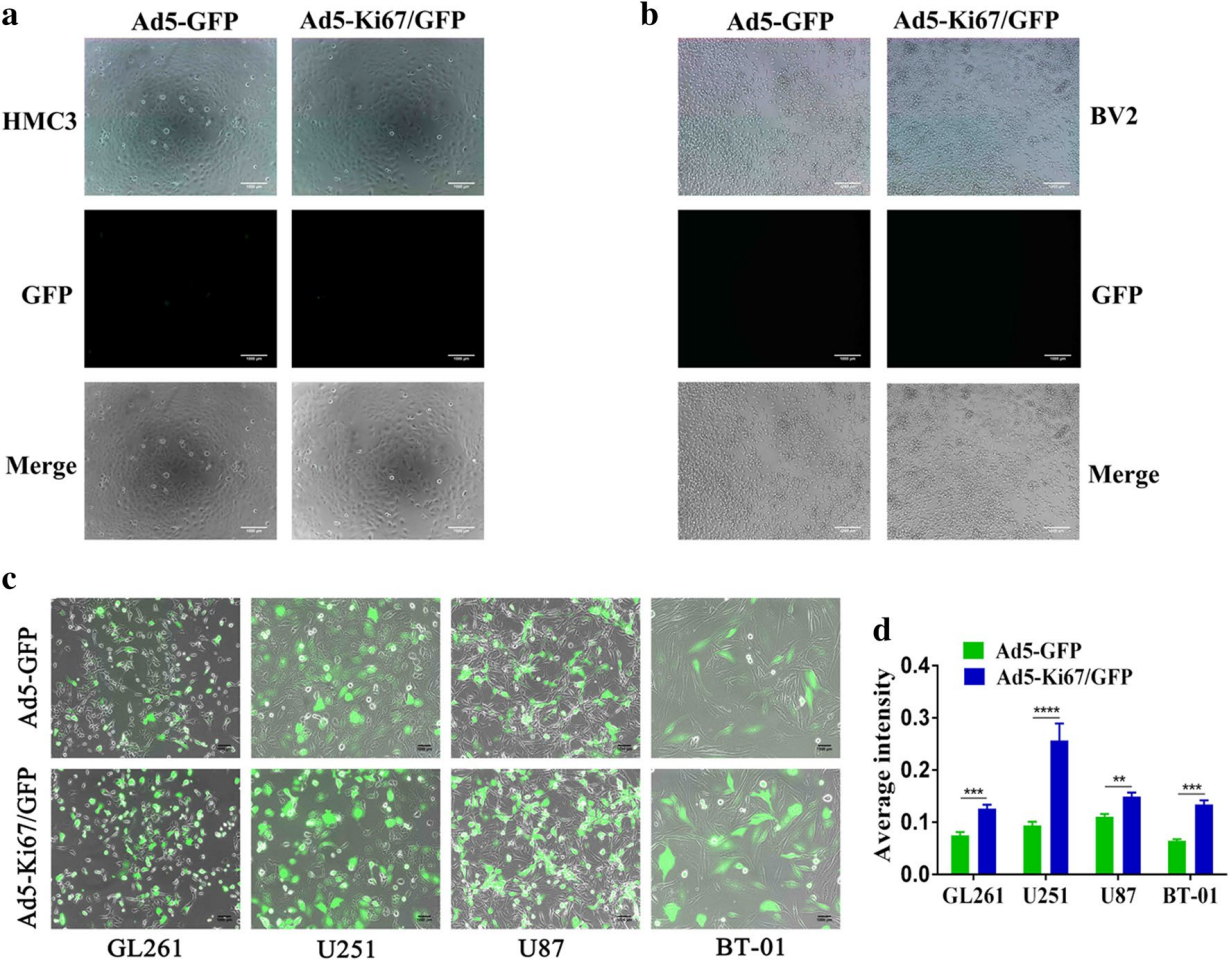
Characteristics of novel constructed oncolytic adenovirus. **a**, **b** Recombinant oncolytic adenovirus (MOI = 40) does not infect microglial HMC3 and BV2 cells (×50, scale bars = 1000 µm). **c** The capacity of recombinant oncolytic adenovirus to infect GBM cells. Representative photomicrographs were obtained from GL261, U251, U87, and primary cells BT-01 that treated with Ad5-GFP and Ad5-Ki67/GFP at an MOI of 40 for 72 h (×50, scale bars = 1000 µm). **d** Quantification of the average intensity difference in GBM cells treated with Ad5-GFP and Ad5-Ki67/GFP for 72 h using Image Pro Plus. (n ≥ 3) *P < 0.05 and **P < 0.01. ***P < 0.001 and ****P < 0.0001

**Fig. 3:**
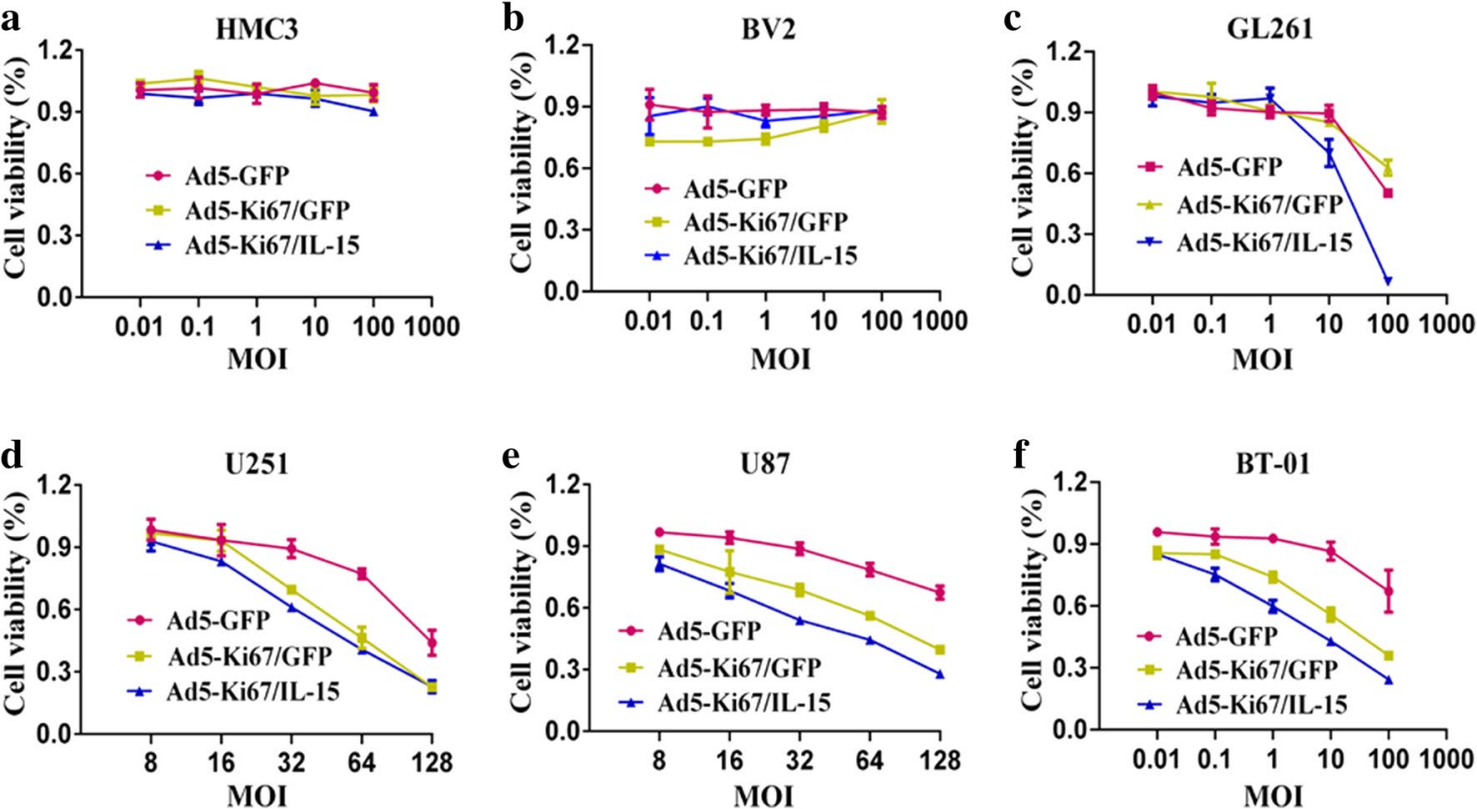
The novel double-controlled OAd driven by the Ki67 core promoter selectively kills GBM cells. **a**, **b** CCK-8 assay was performed to evaluate cell viability of HMC3 and BV2 cells that treated with Ad5-GFP, Ad5-Ki67/GFP, and Ad5-Ki67/IL15 at different MOI. **c**–**f** CCK-8 assay demonstrated that recombinant oncolytic adenovirus inhibited proliferation of GL261, U251, U87, and primary cells BT-01 at different MOI. Inhibition levels were significantly higher in Ad5-Ki67/GFP and Ad5-Ki67/IL15 compared to Ad5. Results are expressed as a percentage of untreated controls

**Fig. 4:**
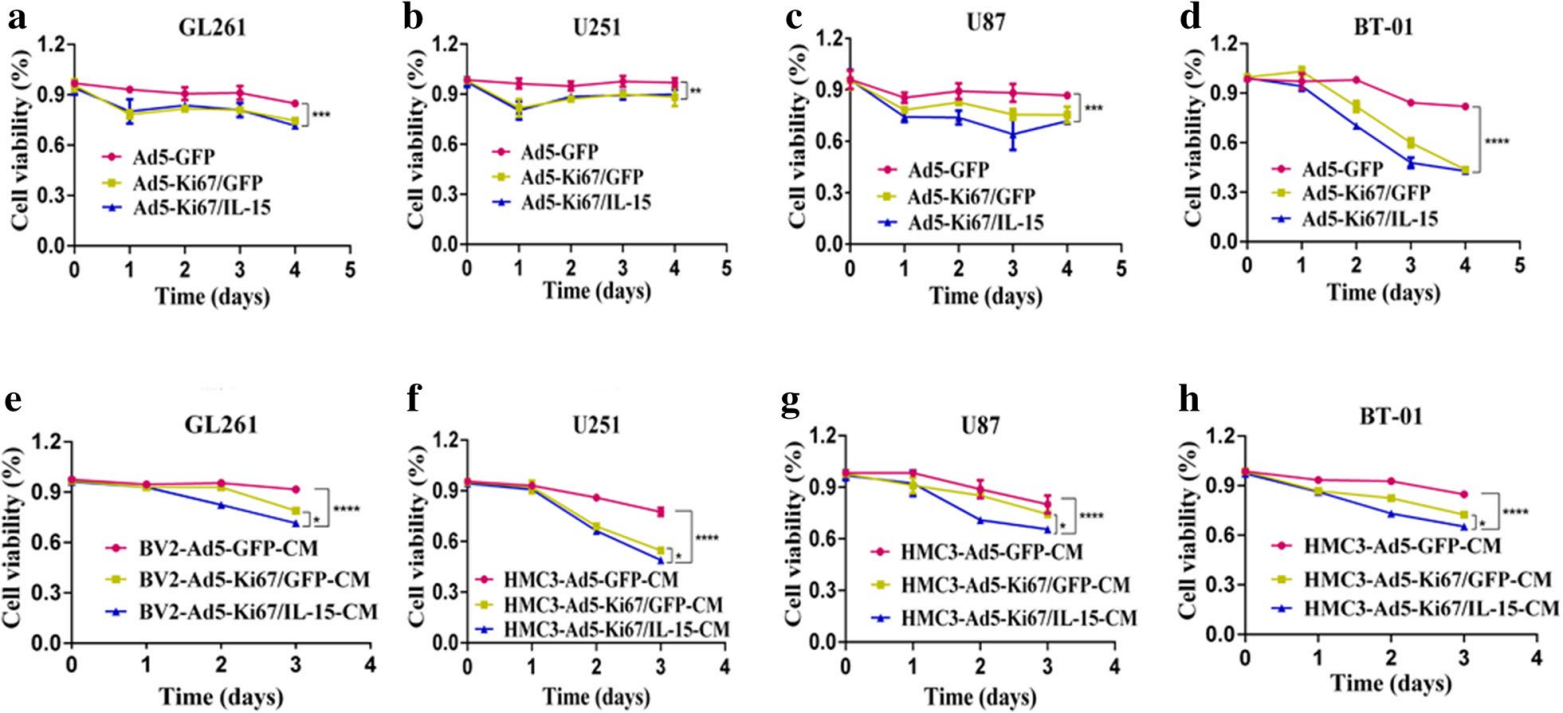
The OAd driven by the Ki67 core promoter and armed with IL-15 enhanced GBM eradication. **a**–**d** CCK-8 assay was performed to evaluate cell viability of GL261, U251, U87 and primary cells BT-01 that treated with Ad5-GFP, Ad5-Ki67/GFP, and Ad5-Ki67/IL15 (MOI = 40). The inhibition ability of Ad5-Ki67/GFP and Ad5-Ki67/IL15 was more potent than Ad5-GFP at different time points. **e** GL261 cell proliferation in response to the conditioned medium from virus-treated BV2 microglial cells (BV2-Ad5-GFP-CM, BV2-Ad5-Ki67/GFP-CM, and BV2-Ad5-Ki67/IL15-CM, MOI = 40) at different time points was determined by CCK8. **f**–**h** U251, U87 and primary cells BT-01 in response to the conditioned medium from virus-treated HMC3 microglial cells (HMC3-Ad5-GFP-CM, HMC3-Ad5-Ki67/GFP-CM, and HMC3-Ad5-Ki67/IL15-CM, MOI = 40) at different time points was determined by CCK8. All results are expressed as a percentage of untreated controls. Data are presented as mean ± SD of three independent experiments. *P < 0.05, **P < 0.01, ***P < 0.001, ****P < 0.0001

A previous study demonstrated that oncolytic viruses controlled under certain promoters could selectively target and kill tumor cells, spreading within the tumor while sparing normal tissue [12]. We found that our recombinant oncolytic adenovirus did not infect normal brain cells, such as HMC3 and BV2 (Fig. 2a, b). GBM cells, including GL261, U251, and U87, and primary cells BT-01 were treated with Ad5-GFP and Ad5-Ki67/GFP and observed using fluorescence microscopy. Our results revealed that the ability of Ad5-Ki67/GFP to infect GBM cells was higher than that of Ad5-GFP, and expression of green fluorescent protein (GFP) in Ad5-Ki67/GFP was significantly enhanced compared to Ad5-GFP (Fig. 2c, d). To evaluate the anti-GBM efficacy of the OAd, experiments were performed using CCK-8. We confirmed that the Ki67 promoter-controlled OAd does not target microglial cells, such as BV2 and HMC3, with normal brain cell viability negligibly affected (Fig. 3a, b). However, we found that the proliferation ability of GBM cells was significantly attenuated when incubated in Ad5-Ki67/GFP and Ad5-Ki67/IL-15 compared to cells incubated with Ad5-GFP at various MOI values (Fig. 3c–f). Subsequently, we also found cell viability gradually decreased in a time-dependent manner, and the anti-GBM efficacy of Ad5-Ki67/GFP and Ad5-Ki67/IL-15 was markedly greater than Ad5-GFP (Fig. 4a–d). These results demonstrated that Ki67 promoter-controlled OAd selectively infects and kills GBM cells while sparing normal cells.

### Ad5-Ki67/IL-15-treated microglia contribute to GBM cell eradication

Microglia cells are immune effector cells inherent in the central nervous system. Tumor-infiltrating microglial cells are the major immune cell population within the GBM microenvironment [28]. With deeper understanding, investigators have found that immunotherapy plays vital roles in the field of GBM treatment. IL-15 is a cytokine that is potentially capable of promoting survival, proliferation, and activation of immune cells. Moreover, we found that Ad5-Ki67/IL-15 prominently attenuated viability in GBM cells compared to Ad5-GFP and Ad5-Ki67/GFP (Fig. 3c–f). To explore IL-15-mediated anti-glioma immunity, we treated HMC3 and BV2 microglia cells using Ad5-GFP, Ad5-Ki67/GFP and Ad5-Ki67/IL-15. Afterwards, CCK8 assay was performed, demonstrating that BV2 treated with Ad5-Ki67/IL-15 significantly inhibited growth of GL261 compared to microglial cells treated with Ad5-GFP and Ad5-Ki67/GFP (Fig. 4e). Furthermore, when U251, U87 and primary cells BT-01 were cultured in conditioned medium from Ad5-Ki67/IL-15-treated HMC3, GBM cell viability was remarkably decreased compared to HMC3 cells treated with Ad5-GFP and Ad5-Ki67/GFP (Fig. 4f–h). These results demonstrated that Ad5-Ki67/IL-15-treated microglia cells enhance anti-GBM efficacy, suggesting that IL-15 is a potential efficacious candidate factor in the field of glioma treatment.

### Oncolytic adenovirus enhances anti-angiogenic capacity

**Fig. 5:**
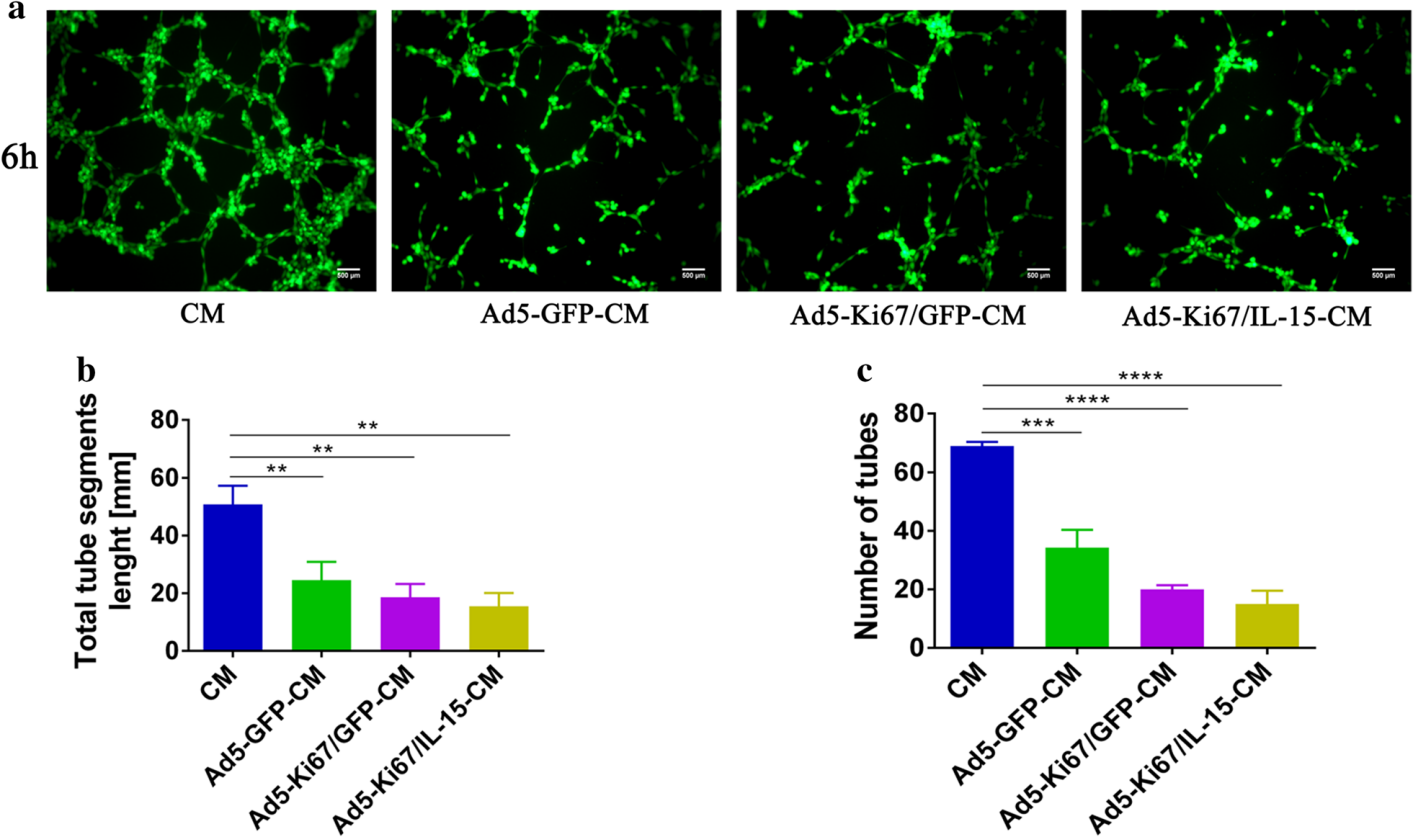
Tube formation capacity of HUVECs incubated in different U251 conditioned media. **a** Glioma cells U251 were respectively treated with Ad5-GFP, Ad5-Ki67/GFP, Ad5-Ki67/IL15 (MOI = 40) for 72 h, and at which time different U251 conditioned media (Ad5-GFP-CM, Ad5-Ki67/GFP-CM and Ad5-Ki67/IL15-CM) were collected. Angiogenic capacity of HUVECs cultured by CM, Ad5-GFP-CM, Ad5-Ki67/GFP-CM and Ad5-Ki67/IL15-CM (MOI = 40) for 6 h on Matrigel (×100, scale bars = 500 µm). CM represented the untreated U251 conditioned medium. **b, c** Total segments length and quantification of number of tubes generated by HUVECs incubated with different conditioned media. *P < 0.05, **P < 0.01, ***P < 0.001, ****P < 0.0001

**Fig. 6:**
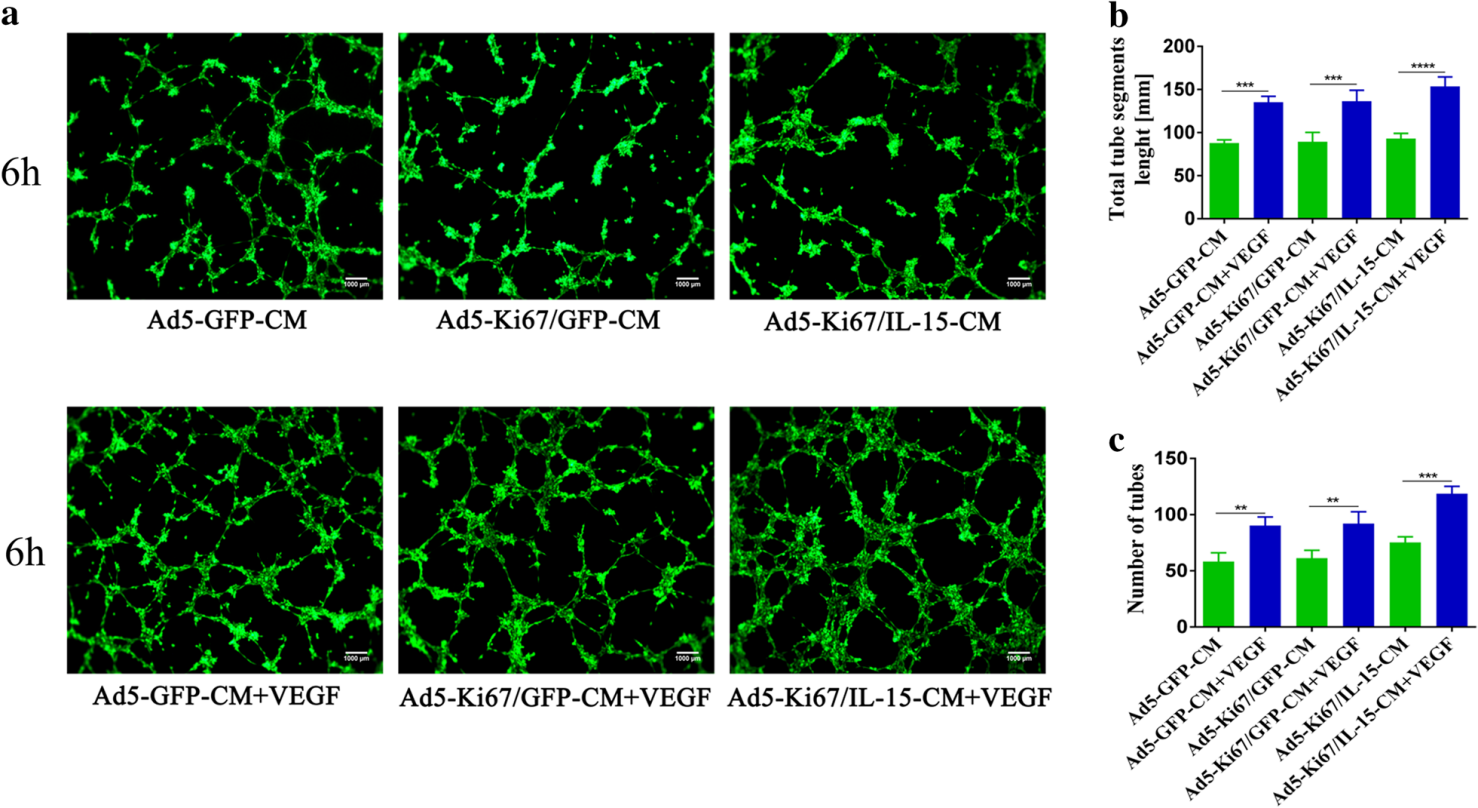
Tube formation capacity of HUVECs incubated in different U251 conditioned media. **a** Angiogenic capacity of HUVECs cultured in Ad5-GFP-CM, Ad5-Ki67/GFP-CM, Ad5-Ki67/IL15-CM, Ad5-GFP-CM + VEGF, Ad5-Ki67/GFP-CM + VEGF and Ad5-Ki67/IL15-CM + VEGF (MOI = 40) for 6 h on Matrigel (×100, scale bars = 500 µm). **b, c** Total segments length and quantification of number of tubes generated by HUVECs incubated with different culture conditions. *P < 0.05, **P < 0.01, ***P < 0.001, ****P < 0.0001

Angiogenesis is of great importance in glioma progression and has increasingly become a research hotspot in the field of GBM treatment. Previous data revealed that oncolytic herpes simplex virus (HSV)-mediated decreases in angiogenesis might be due to either viral replication or the inflammatory response [29]. Thus, we focused on studies of the anti-angiogenic effects of our oncolytic adenovirus. In vitro, we found that tube formation capacity at 6 h was significantly decreased in HUVECs treated with different viruses for 72 h (Ad5-GFP, Ad5-Ki67/GFP and Ad5-Ki67/IL15) compared to their untreated counterparts (Additional file 2: Fig. S2). Meanwhile, our results also revealed that tube formation at 6 h was more lower in HUVECs incubated with different U251 conditioned media (Ad5-CM, Ad5-Ki67-CM and Ad5-Ki67/IL15-CM) compared to the control group (Fig. 5a). Furthermore, tube segment lengths and the number of tubes in the groups with OAd were significantly reduced compared to controls (Fig. 5b, c). When VEGF (6 ng/ml) was added to different U251 conditioned media and imaged at 6 h, we found that media with VEGF contributed to enhanced angiogenic activity compared to control groups (Fig. 6a), and total tube segment lengths and number of tubes were also quantified (Fig. 6b, c). These data demonstrated that OAd exerts powerful anti-angiogenic activity.

### Secretion of VEGF in GBM cells

**Fig. 7:**
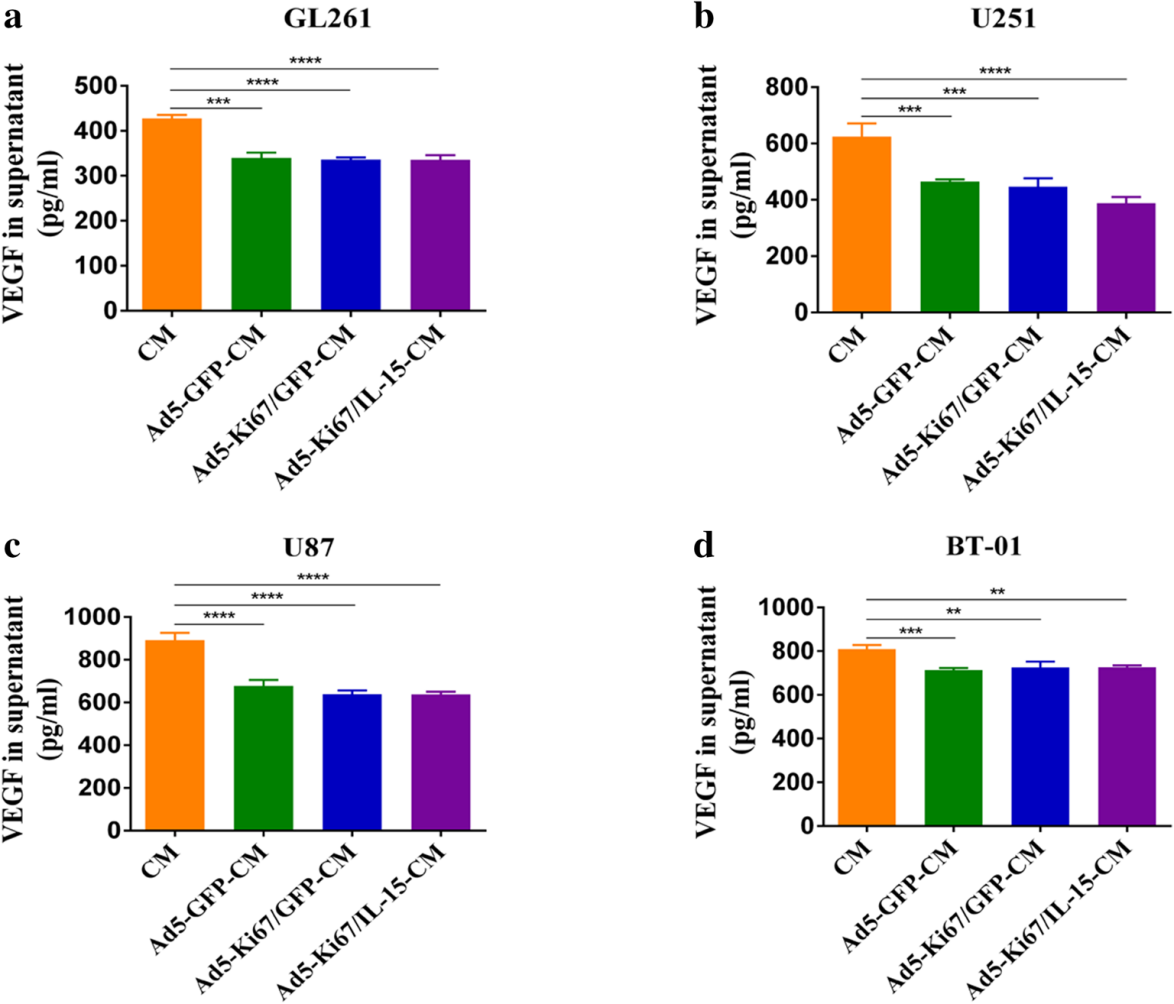
VEGF levels in different glioma conditioned media measured by ELISA. **a** VEGF levels were significantly lower in Ad5-GFP-CM, Ad5-Ki67/GFP-CM, and Ad5-Ki67/IL15-CM (MOI = 40) than in untreated conditioned medium in GL261 cells. **b** U251. **c** U87. **d** Primary cells BT-01. *P < 0.05, **P < 0.01, ***P < 0.001, ****P < 0.0001

VEGF is a crucial cytokine for stimulating angiogenesis of vascular endothelial cells and maintaining the stability of tube structure. Prior studies confirmed that VEGF secreted by tumor cells contributes to promoting tumor angiogenesis [30]. To examine the effect of viral infection on VEGF production in glioma cells, ELISAs were performed on conditioned media collected from GL261, U251, and U87 glioma cells and primary cells BT-01 after infection with OAd at a certain multiplicity of infection. Conditioned media not treated with adenovirus were also obtained. We observed that VEGF levels in the Ad5-CM, Ad5-Ki67-CM and Ad5-Ki67/IL15-CM treated groups from GL261, U251, and U87 glioma cells and primary cells BT-01 were significantly decreased (Fig. 7), indicating that OAd may inhibit angiogenesis by attenuating VEGF secretion from glioma cells.

## Discussion

It is well known that oncolytic adenoviruses are emerging agents in the field of GBM therapeutics and can regulate the GBM immune microenvironment [31]. Numerous studies have shown that immunosuppression in GBM results from downregulation of MHC I, expression of immunosuppressive factors, inhibition of T cell proliferation, and recruitment and M2-like polarization of macrophages [32–34]. Macrophages/microglia are typically activated to undergo polarization into opposite phenotypes, classically activated M1, which is associated with inflammation and immunity, and alternatively activated M2, which is associated with repair and immune suppression [35]. In GBM, microglial cells are the most prevalent immune cells and are predominantly M2-like [28]. To overcome the bottleneck of GBM treatment, we developed a novel double-controlled oncolytic adenovirus driven by the Ki67 core promoter and armed with IL-15. For the first time, we demonstrated that this strategy is both efficacious and specific in GBM cells. These findings provide a solid foundation for us to explore the oncolytic effects in vivo and their specific mechanisms in the future.

Immune factors have been tested in combination with oncolytic viruses in several GBM models and have been shown to improve anti-glioma activity. Design of a Phase I clinical trial was recently completed that combined intratumoral FDA-approved oHSV with IL-12 in recurrent or progressive malignant gliomas, demonstrating both efficacy and safety [36]. The unique immune characteristics and additional safety issues associated with therapy in the brain complicate combination approaches using virus. The dual effects of GBM-targeting oncolytic viruses and immune-modulators have been much more effective than virus alone in preclinical tumor models, including in IL-12 secreting HSV and ADV armed with IL-4 for glioma, as well as in clinical trials [9, 36, 37]. However, preclinical immunotherapy studies in GBM have been hampered by immune suppression, tolerance and invalid activation.

There is currently limited efficacy of GBM immunotherapy. High mutational load with increased neo-antigens correlates strongly with immunotherapy efficacy in patients [37], while those with low mutational loads, such as GBM, exhibit little response [38]. However, GBM has better responses to oncolytic viral therapy, although many patients also respond to standard care [3]. Previous studies demonstrated that engineered OAd elicited good response in GBM [8, 39]. IL-15 was an effective immunologic mediator, and it induced activation and survival of effector immune cells, which were indispensable for its antitumor immunity activity. Immune cells stimulated with IL-15 produce high levels of IFN-γ and TNFα [23], and IL-15’s strong immune-stimulatory activity coupled with an apparent lack of toxicity made it a promising candidate for tumor therapy.

Adenoviral E1A is necessary and plays an important role in the process of viral infection and replication [12]. The Ki67 promoter regulates transcription of the E1A gene, and IL-15 was inserted into the viral genome. The dual effect of Ki67 promoter-regulated OAd and IL-15 contributed to the eradication of GBM cells. Importantly, dual therapeutic methods exhibit a lasting inhibitory effect. This synergy may antagonize the side effects of GBM immunosuppression to potentially enhance oncolytic efficacy. A growing number of studies have described immune cell-mediated glioma destruction, including indirect killing of glioma cells by T cell, NK cell and macrophages induction [37, 40]. Ad5-Ki67/IL-15 contributed to inducing microglial-mediated anti-GBM effects, possibly because IL-15 induced IFN-γ or TNF-α production, which further increased the dual combination therapy due to stimulation of immune cells. IFN-γ signals in the tumor microenvironment can skew macrophage polarization from M2 to M1-like, with tumor-suppressive and anti-angiogenic properties [41].

Activation of microglial cells apparently promoted treatment effects in brain tumor-initiating cells, and the treatment-mediated increased efficacy in tumor suggests a key role for microglial cells [42]. Previous studies revealed that microglia factors stimulated the apoptosis of glioma cells, and the toll-like receptor 3 agonist poly caused microglia to secrete factors that killed glioma cells [42, 43]. Moreover, the stimulation microglia by intratumoral injection of lipopolysaccharide reduced glioma growth in mice [44]. We found that microglia, effector immune cells from normal brain tissue, amplified the effect of dual combination therapy. This might be due to activation of microglia, through polarization into M1-like cells and secretion of anti-tumor factors, suggesting that oncolytic virus combined with immunotherapy might be more clinically beneficial.

GBMs are highly vascularized, and the extent of angiogenesis is significantly correlated with their prognosis [45]. The potent capacity of GSCs or MSCs to generate vascular pericytes allows active vascularization in GBMs to support tumor growth [30, 46]. The attachment of pericytes to endothelial cells (ECs) contributes to maintain and stabilize capillary-like structures [30]. Prior studies revealed that HSV-1 inhibits angiogenesis by directly infecting and disrupting ECs, which suppresses tumor growth [29], while we found that OAd showed anti-angiogenic capacity by reducing VEGF levels in GBMs. Targeting angiogenesis continues to be an attractive therapeutic modality in GBMs, and these findings may provide new therapeutic insight for GBM patients in the future.

## Conclusions

We demonstrated that double-controlled oncolytic adenovirus driven by the Ki67 core promoter and armed with IL-15 efficiently improved the immune microenvironment and generated potent oncolytic efficacy against GBMs, and the effect was more targeted and specific to GBMs. We also provided evidence that viral-induced anti-angiogenic properties might impede GBM progression. These results provide new insights into possible future treatment options utilizing specific promoter-controlled OAd and expressing IL-15 in GBM patients.

## Supplementary information

**Additional file 1: Fig. S1.** Schematic diagram of recombinant oncolytic adenovirus construction. In Ad5-Ki67/GFP, the type 5 adenovirus E1A promoter was replaced with the Ki67 promoter, enabling the oncolytic adenovirus to replicate in GBM cells that expressed Ki67. In Ad-Ki67/IL15, based on Ad5-Ki67/GFP construction, the hIL-15 gene expression box was inserted into the E3 gene region (including the CMV promoter, IL-15 gene, and SV40 PolyA), so that the virus expressed the IL-15 gene when targeting glioblastoma cells.

**Additional file 2: Fig. S2.** Tube formation capacity of HUVECs treated with different oncolytic adenoviruses. a. Angiogenic capacity of HUVECs treated with Ad5-GFP, Ad5-Ki67/GFP, and Ad5-Ki67/IL15 (MOI = 40) on Matrigel (× 100, scale bars = 500 µm). b. Quantification of number of tubes, total segment length generated by HUVECs treated in different oncolytic adenovirus (n ≥ 3) *P < 0.05, **P < 0.01, ***P < 0.001, ****P < 0.0001.

## Data Availability

The datasets used and analyzed during the current study are available from the corresponding author on reasonable request.

## Abbreviations

GBM: Glioblastoma
OAd: Oncolytic adenovirus
Ad5: Adenovirus type 5
IL-15: Interleukin-15
MOI: Multiplicity of infection
HUVEC: Human umbilical vein endothelial cell
HSV: Herpes simplex virus
VEGF: Vascular endothelial growth factor
ECs: Endothelial cells
