# Supplementary figures and images for: Efficacy of a novel double-controlled oncolytic adenovirus driven by the Ki67 core promoter and armed with IL-15 against glioblastoma cells
Source: Cell Biosci. 2020 Oct 27;10:124. doi: 10.1186/s13578-020-00485-1 (PMC7592588; doi:10.1186/s13578-020-00485-1)

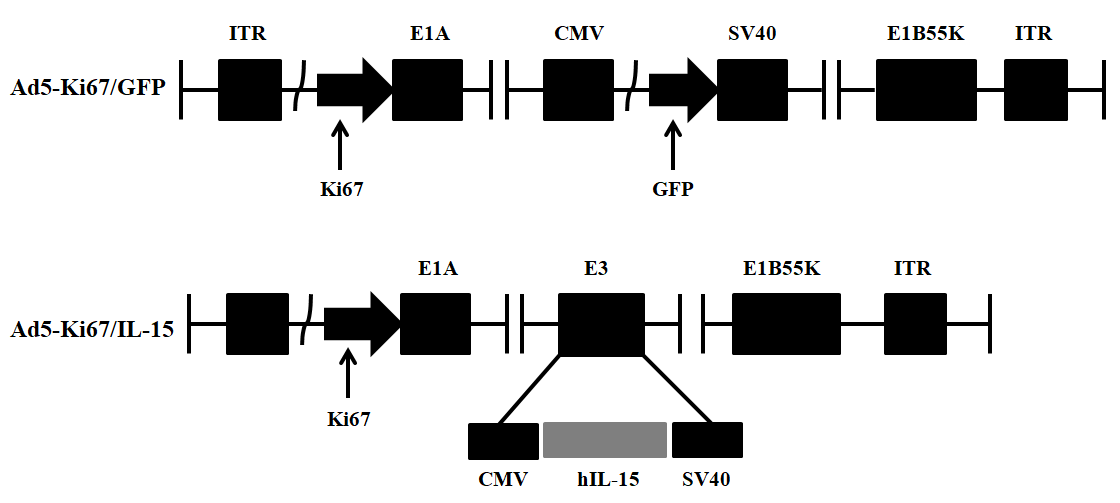

Supplement: Supplementary file 1 — Additional file 1: Fig. S1. Schematic diagram of recombinant oncolytic adenovirus construction. In Ad5-Ki67/GFP, the type 5 adenovirus E1A promoter was replaced with the Ki67 promoter, enabling the oncolytic adenovirus to replicate in GBM cells that expressed Ki67. In Ad-Ki67/IL15, based on Ad5-Ki67/GFP construction, the hIL-15 gene expression box was inserted into the E3 gene region (including the CMV promoter, IL-15 gene, and SV40 PolyA), so that the virus expressed the IL-15 gene when targeting glioblastoma cells. [file 13578_2020_485_MOESM1_ESM.tif]

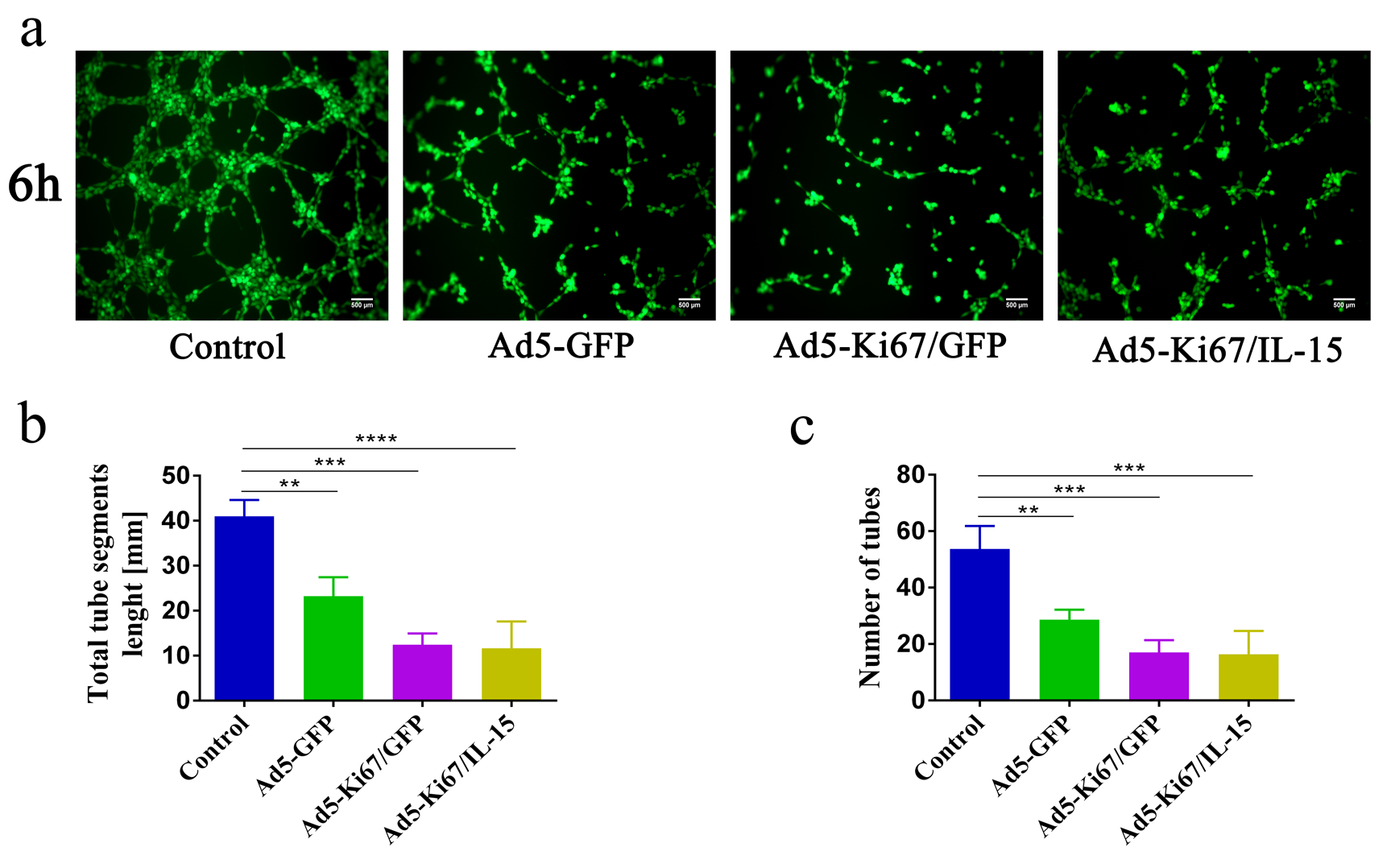

Supplement: Supplementary file 2 — Additional file 2: Fig. S2. Tube formation capacity of HUVECs treated with different oncolytic adenoviruses. a. Angiogenic capacity of HUVECs treated with Ad5-GFP, Ad5-Ki67/GFP, and Ad5-Ki67/IL15 (MOI = 40) on Matrigel (× 100, scale bars = 500 µm). b. Quantification of number of tubes, total segment length generated by HUVECs treated in different oncolytic adenovirus (n ≥ 3) *P < 0.05, **P < 0.01, ***P < 0.001, ****P < 0.0001. [file 13578_2020_485_MOESM2_ESM.tif]
